# Hexahedral Localization (HL): A Three-Dimensional Hexahedron Localization Based on Mobile Beacons

**DOI:** 10.1155/2013/965138

**Published:** 2013-11-11

**Authors:** Linlan Liu, Haili Zhang, Xiaotian Geng, Xin Shu

**Affiliations:** ^1^School of Information Engineering, Internet of Things Technology Institute, Nanchang Hangkong University, Nanchang 330063, China; ^2^Tianjin Baodi No. 1 Middle School, Tianjin 301800, China; ^3^School of Software, Internet of Things Technology Institute, Nanchang Hangkong University, Nanchang 330063, China; ^4^School of Electrical Engineering and Computer Science, Washington State University, Pullman, WA 99163, USA

## Abstract

In wireless sensor networks, localization is one of the fundamental technologies and is essential to its applications. In this paper, we propose a three-dimensional range-free localization scheme named hexahedral localization. In the scheme, the space is divided into a lot of hexahedrons. Then, all the unknown nodes are located by utilizing the perpendicular properties of the trajectory. The contribution of our scheme can be summarized into two points. First, it fills the gap of shortage of three-dimensional localization based on mobile beacons. Second, it brings in the outstanding localization accuracy. The simulation result reveals that this localization scheme has the relative high accuracy. At the end of the paper, the performance and error of our scheme are analyzed in aim of improving in the future work.

## 1. Introduction

Wireless sensor network (WSN) is considered as one of the most influencing technologies in the 21st century and one of the inventions which would change the future world [1]. As the technologies of sensor, microsystem, wireless communication, and the computer developed, the wireless sensor networks are applied more and more widely. In WSN, the location of nodes is significant to the detection. Location information also supports many fundamental network services, including network routing, topology control, coverage, boundary detection, and clustering [2]. So, it is obvious that localization is essential to the applications of wireless sensor network. The localization mechanisms in WSN are usually classified into two categories: range-based mechanisms and range-free mechanisms [1]. Typical range-free localization algorithms include Centroid [3–5], APIT [6–11], and DV-HOP [12–14]. They leverage the limited hardware to acquire the location of the nodes with the advantage of low cost and little environmental impact. On the other hand, the range-based mechanisms, such as TOA [15, 16], TDOA [17–20], and RSSI [21–25], utilize signal or acoustic wave to get the distance or orientation between nodes in order to calculate the nodes' coordinate.

To sum up, most of the localization mechanisms employ beacon (anchor) nodes and utilize the relationship between the beacons and the unknown node to gain the location of the nodes. However, the beacons should be embedded with the GPS which leads to high hardware cost. In light of this, the researchers propose the localization schemes based on mobile beacons in aim of reducing the cost of hardware. The localization based on mobile beacons utilizes just a few beacons broadcasting the message while moving among the unknown nodes instead of deploying many static beacons. These methods cut down the overhead by a wide margin.

The rest of the paper is organized as follows.  Section 2 introduces the related works about the mobile beacon based localization in the past decade.  Section 3 gives out the design of the HL.  Section 4 is the simulation and result, followed by Section 5 which analyzes the relationship among the parameters. At last, Section 6 represents the error analysis, and we conclude in Section 7.

**Figure:**
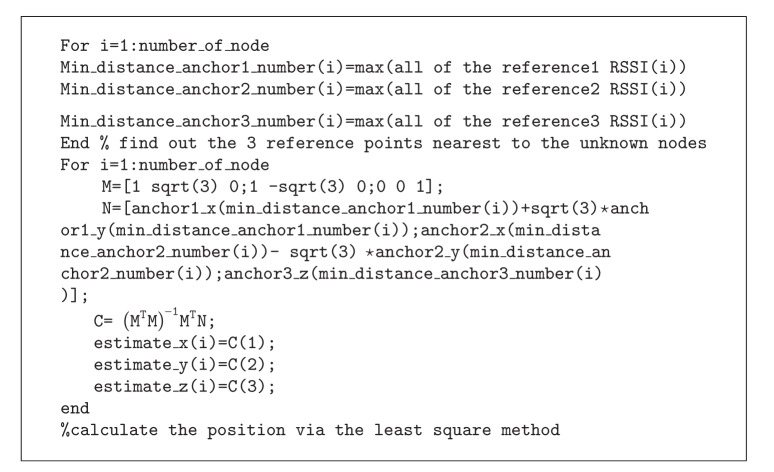
PSEUDOCODE 1

## 2. Related Works

During the past decade, the localization schemes based on mobile beacons have been developed in a variety of directions.

The pioneer work can be traced back to 2004. North Carolina State University [26] firstly depicts the initial model of the mobile beacon-based localization. They acquire the node's location via the PDF (probability distribution function) of the estimated position according to the RSSI (received signal strength indicator). Then, they make some remarks regarding two properties that the trajectory should have. At last, the experimental results reveal an unexpectedly good accuracy, almost an order of magnitude better than other static approaches. In 2005, the researcher of National Cheng Kung University, Ssu et al. [27], selects more than 3 beacon points to determine the position of unknown nodes. In the paper, the scheme adopts the RWP (random waypoint), and the paper analyzes the accuracy under different radio ranges of beacon moving speed, and so forth. The result reveals that, as a range-free approach, its accuracy is competitive to other range-based approaches. In 2006, the authors of [27] propose a new localization algorithm based on aerial beacons [28]. It utilizes an aerial beacon moving upon the sensor nodes to locate them via the geometry principles. It is a three-dimensional localization algorithm, although it is used in the two-dimensional environment. The simulation shows that its performance is better than other range-free localization schemes. In 2007, Purdue university [29] compared three trajectories of the beacons' movement named Scan, Double Scan, and Hilbert. The result of simulation describes that Scan has the lowest localization error among the three trajectories, followed closely by Hilbert. However, Hilbert is the most robust to the obstacles. In 2008, the Chinese Academy of Science [30] improves the algorithm in [27] by searching for the “maximum RSSI” point as the midpoint of the chord. It gets more reliable reference points to make the accuracy more precise by the ratio of 50%. In 2009, the Chinese academy of Science [31] further improves the previous scheme through searching for 4 reference points to ensure the position of the unknown nodes. Compared to the previous work, the proposed approach enhances accuracy to a certain degree. In the same year, the Gwangju Institute of Science and Technology [32] improves the algorithm of [27] with the geometric constraints. It points out that the selection of the reference point in [27] is inaccurate and selects 3 noncollinear reference points to locate the nodes with the geometric constraints. As a result, the accuracy is improved. The Chinese National University of Defense and Technology [33] proposed two algorithms on the path planning. These two algorithms are based on graph theory and are called breadth-first and backtracking greedy. The goal of path planning is locating the nodes within less time and cost. In the view of coverage and cost, these two algorithms are effective. In addition, they obtain higher precision and are robust in the environment of the nodes randomly deployed. In 2010, the Ocean University of China [34] proposes a novel localization algorithm based on the mobile beacon. It plans a regular path consisting of equilateral triangles and utilizes the geometric property to locate the unknown nodes. As a latecomer in the part representation arena, this scheme attracts people's eyes. Its design is motivated by the phenomenon between RSSI and straight trajectory of the mobile beacon. The experiment of the scheme with 100 TELOSB motes proves that this scheme is superior to all the existing approaches in terms of high precision. 

In 2011, Ou [35] proposes a range-free localization scheme using mobile anchor nodes equipped with four directional antennas. In the proposed approach, each mobile anchor node determines its position via GPS, and then broadcasts its coordinates as it moves through the WSN. The sensor nodes detect these beacon messages and utilize a simple processing scheme to determine their own coordinates based on those of the anchors. It removes the requirement for specific ranging hardware on the sensor nodes and avoids the need for communications between the sensor nodes. In 2012, the INRIA [36] proposed a novel DeteRministic dynamic bEAcon Mobility Scheduling (DREAMS) algorithm, without requiring any prior knowledge of the sensory field. In this algorithm, beacon trajectory is defined as the track of depth-first traversal (DFT) of the network graph, which thus is deterministic. The mobile beacon performed DFT dynamically, under the instruction of nearby sensors on the fly. It moved from sensor to sensor in an intelligent heuristic manner according to received signal strength (RSS) based distance measurements. It was proved that DREAMS guarantees full localization (every sensor is localized) when the measurements are noise free. In the same year, Chang et al. [37] proposed the first study that applies the mobile anchor to improve the location inaccuracy under the condition that all sensors are with different sizes of estimative regions. In 2013, a range-free localization mechanism with ring overlapping by utilizing mobile anchors was proposed by Chen et al. [38]. Since the mobile anchor and the reference node know their own locations, the B-rings, in which the blind node is located, can be precisely derived. Therefore, by overlapping the B-rings, the proposed mechanism can obtain good location estimation for the blind node. Besides, two movement schemes, BTS and ESS, for mobile anchor are also proposed. The proposed scheme has better accuracy than other existing related schemes including ROCRSSI scheme, Centroid scheme, and PBCC scheme.

However, we see that almost all of the algorithms above rarely refer to the three-dimensional localization, since the localization in three-dimensional environments is more complex. According to this blankness, this paper proposes a novel three-dimensional localization scheme based on mobile beacon called HL (hexahedral localization). It is able to locate without any additional hardware and reach the relative high accuracy.

## 3. The Design of HL

In this section, the train of thought about the HL is described. The design of HL is inspired by literature [34]. Firstly, the experiment on RSSI versus Distance is made. Then, we present our new scheme. At last, we optimize the scheme.

### 3.1. The Experiment of RSSI versus Distance

As the most popular parameter used in the localization process, RSSI has the advantage of low cost and convenient operation. Theoretically, RSSI obeys the following formula [39]:

(1) PL(d)=−32.44−20log⁡fc−20log⁡d.

PL(*d*) is the RSSI according to the distance of *d*, and *f*_*c*_ is the carrier frequency. From the formula above, we can get that the RSSI decreases as *d* increases.

**Figure 1:**
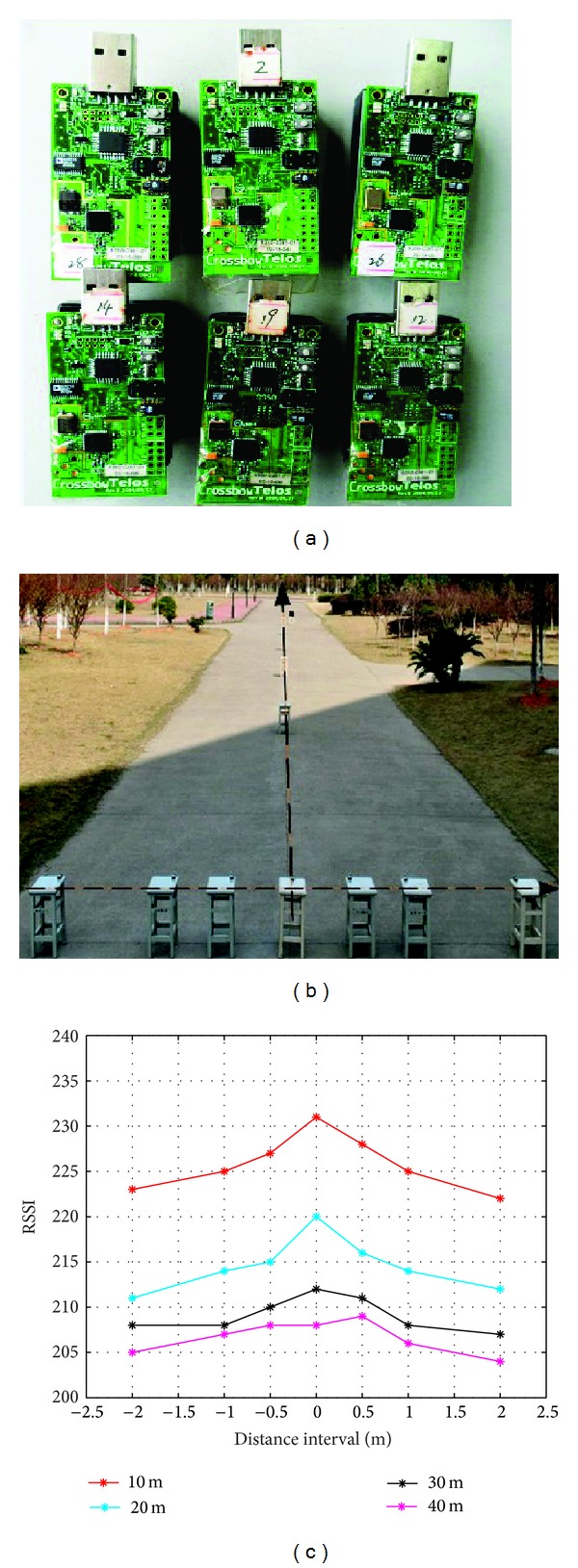
The experimental environment and result.

Similar literature [34], we observe the interesting regularity. As shown in Figure 1, we deploy 11 TELOSB motes on the campus to observe the RSSI that the node on longitudinal axis receives from which is on the transverse axis. We are surprised to find that the data could plot into a curve as shown in Figure 1. The only difference between our research and literature [34] is the length of the transverse axis. In fact, it is unnecessary to study the width which is too large for the inaccurate RSSI.

As Figure 1 shows, we assume the transverse axis as the trajectory of a mobile beacon. When the mobile beacon tracks along a straight line, the nearest point of the unknown node is the foot point of the trajectory. At the same time, the RSSI is the largest.

Different from literature [34], the purpose of our experiment is to get the suitable distance that the RSSI is available. From Figure 1, we can obtain the trusty distance is 30 m when the radius is about 50 m. And we continue to do the similar but more accurate experiment under the radius from 40–100 m by the step of the 10 m. The result is shown in Table 1. 

According to Table 1, the trusty radius analogously equal to 60% of the radius.

### 3.2. The Model of the HL

**Figure 2:**
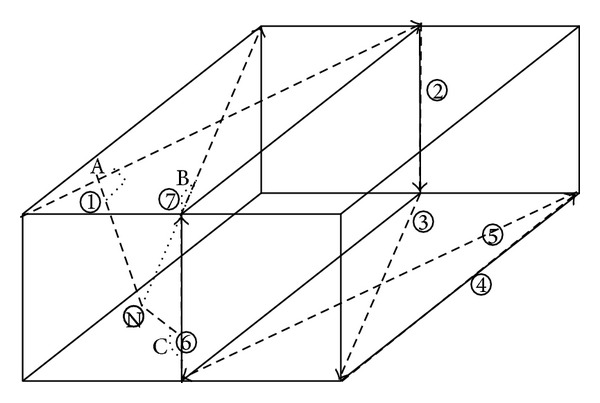
The model of HL.

The observation above motivates the design of HL. We project HL as shown in Figure 2.

The mobile beacon moves along the given trajectory and broadcasts its own position periodically. Via RSSI, the unknown node selects the nearest reference points on the trajectory. The coordinate of the reference point is

 (*x*_*a*_, *y*_*a*_, *z*_*a*_)(*x*_*b*_, *y*_*b*_, *z*_*b*_)(*x*_*c*_, *y*_*c*_, *z*_*c*_) .

And the unknown node is

 (*x*, *y*, *z*).

The direction vector of *①*, *⑦*, *⑥* is given as

 (*i*_1_, *j*_1_, *k*_1_)(*i*_2_, *j*_2_, *k*_2_)(*i*_3_, *j*_3_, *k*_3_).

Then, we have the following equations:

(2) i1(x−xa)+j1(y−ya)+k1(z−za)=0i2(x−xb)+j2(y−yb)+k2(z−zb)=0i3(x−xc)+j3(y−yc)+k3(z−zc)=0⇒M(xyz)=N,M=(i1j1k1i2j2k2i3j3k3),  N=(i1xa+j1ya+k1zai2xb+j2yb+k2zbi3xc+j3yc+k3zc).

According to the least square method,

(3) $$\begin{array}{c} \left(\begin{array}{c} x\\ y\\ z \end{array}\right)={\left(M^{T}M\right)}^{-1}M^{T}N. \end{array}$$

There must be a lot of errors in the process of calculation via the least square method. We will analyze that in Section 6.

### 3.3. The Optimization of the Trajectory

**Figure 3:**
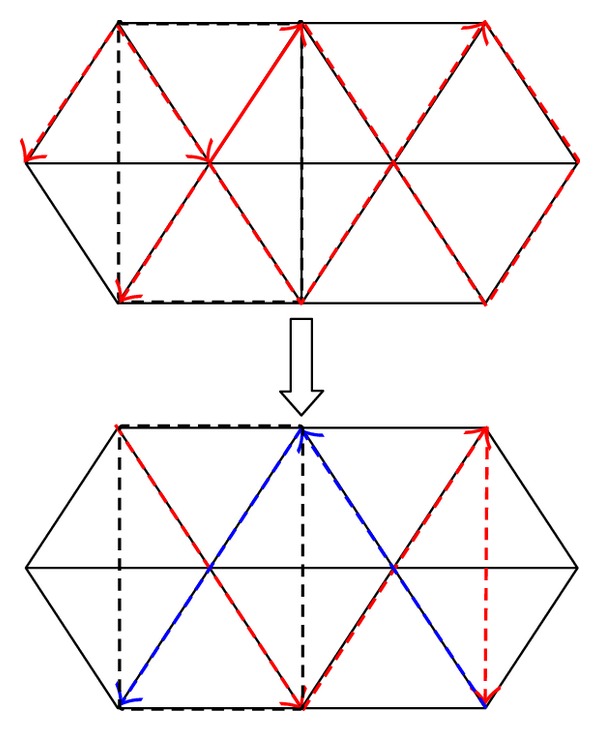
The transformation of the two-dimensional trajectory.

We have given out the trajectory of the HL. But how to make the model reasonable is an important issue. From literature [34], it can be known that the equilateral triangle is the best trajectory on the two-dimensional flat. And interestingly, we find that the equilateral triangles can be connected and divided into rectangles like Figure 3. The trajectory we proposed is more controllable at the same time.

As the trajectory described in Section 3.2, according to the radius *R*, the ratio of the hexahedron's edges should be optimized.

**Figure 4:**
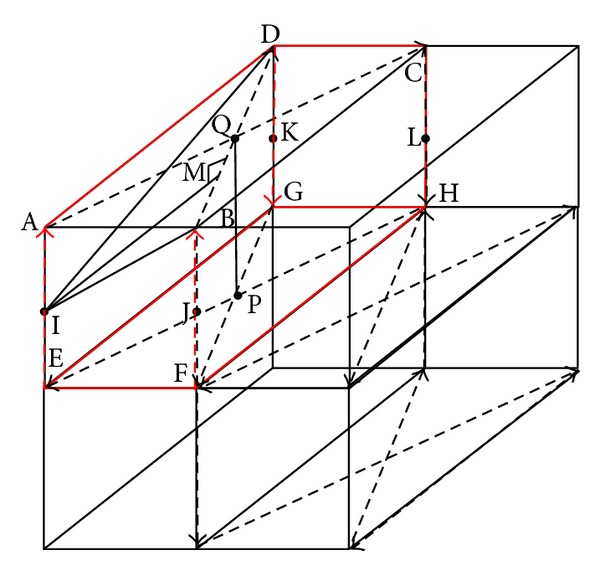
Example of the hexahedron.

In Figure 4, take the red hexahedron, for example, AC, BD, HE, GF, AE, BF, DG, and CH are all the trajectories of the mobile beacon. To ensure the coverage of mobile beacons, the extension that the signal propagates should be equal. In another word, the point in the hexahedron which is furthest from the trajectory should be covered in the extension. According to the trusty radius, the largest distance should be equal to the trusty radius. As the trajectories are deployed symmetrically as shown in Figure 4, we find that the points I, J, K, L are the furthest points to the bevel trajectory like AC, BD, HE, and GF. And the point on PQ is the furthest point to the vertical trajectory like AE, BF, DG, and CH. We can calculate the distance between the furthest point and the trajectory

(4) IM=AE24+AB2AD2AB2+AD2

And the distance from PQ to the trajectory AE, BF, DG, and CH is

(5) BQ=AB2+AD22.

The distance IM and BQ should be equal to the trusty radius. Then,

(6) $$\begin{array}{c} R_{\text{trusty}}=\sqrt{\frac{{\text{AE}}^{2}}{4}+\frac{{\text{AB}}^{2}{\text{AD}}^{2}}{{\text{AB}}^{2}+{\text{AD}}^{2}}}=\frac{\sqrt{{\text{AB}}^{2}+{\text{AD}}^{2}}}{2}. \end{array}$$

As described in literature [34], the ratio of AB and AD should be $1\text{ : }\sqrt{3}$. According to (6),

(7) $$\begin{array}{c} R_{\text{trusty}}\text{ : AE : AB : AD}=\text{1 : 1 : 1 : }\sqrt{3}. \end{array}$$

The whole process of localization can be described as follows.

**Step 1**

The unknown nodes are randomly deployed in the three-dimensional space.

**Step 2**

The anchor moves along the trajectory according to the radius and broadcasts its location information and ID.

**Step 3**

The nodes receive and record information from the anchor (including signal strength).

**Step 4**

Each node finds out the nearest reference points on each of the three kinds of trajectory according to the RSSI.

**Step 5**

Implement the HL and calculate estimated position.

**Step 6**

The localization is finished.

**Figure 5:**
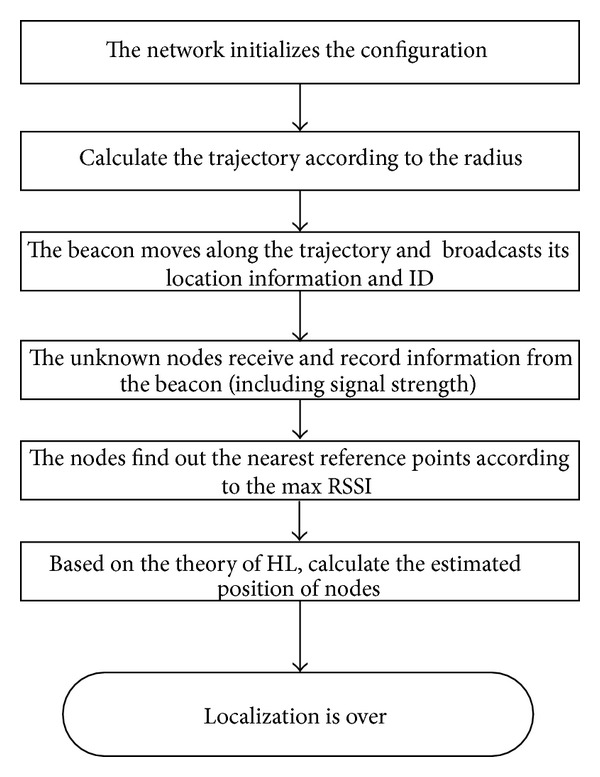
The flow chart of HL.

The flow chart of IAPIT-3D is shown in Figure 5.

The Matlab pseudocode of localization period is displayed in Pseudocode 1.

## 4. Simulation and Result

In order to verify the theoretical feasibility of HL, the scientific tool MATLAB is adopted for the simulation. Take the conclusion of Section 3.1 for premise, we deploy 400 unknown nodes in the space of size 100 m × 100 m × 100 m. The experiment is separately simulated by the moving step length of 1 m, 2 m, 3 m, 5 m, 6 m, 10 m, and 15 m. The result is described by the average absolute error and normalized average error (which is normalized to the ratio of the absolute average error to the radio range).

**Figure 6:**
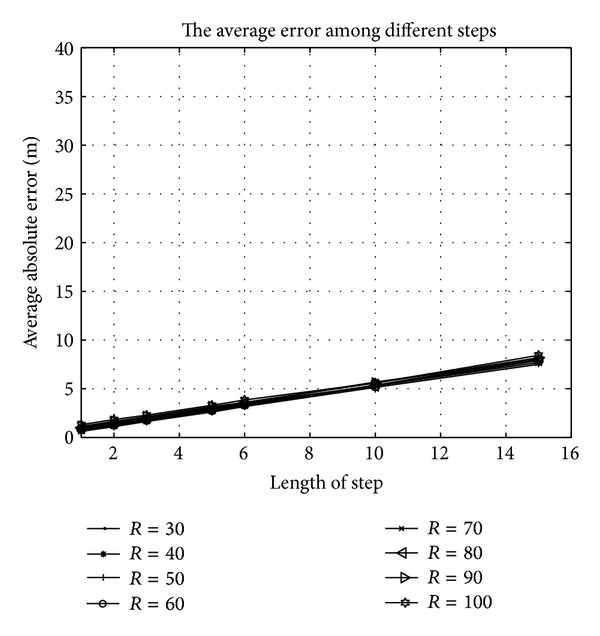
The average absolute error under different moving step lengths.

**Figure 7:**
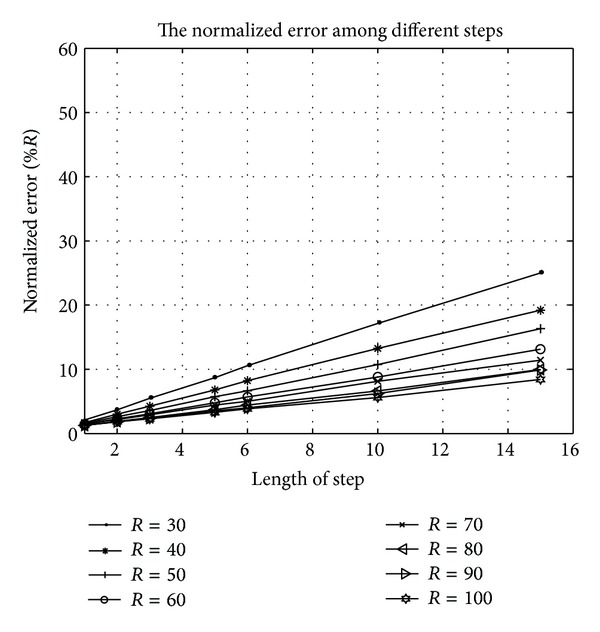
The average normalized error under different moving step lengths.

As Figures 6 and 7 show, the errors are increasing as the moving step length increases. In another word, the longer the length of step is, the more inaccurate the localization is. The reason is obvious that the shorter the step, is the more virtual beacon nodes are deployed. The location error is shown in Tables 2 and 3.

## 5. The Relationship among the Parameters

### 5.1. The Radius and the Absolute Error

**Figure 8:**
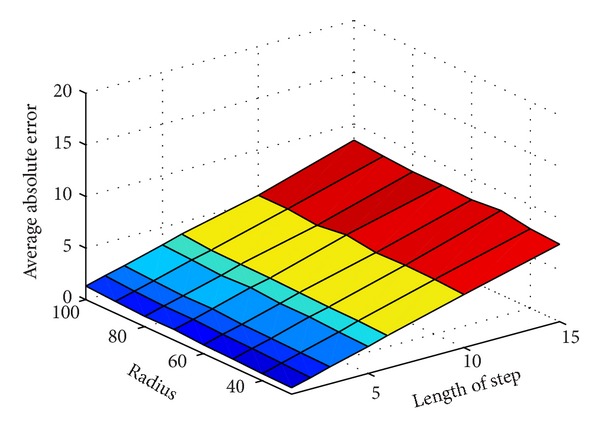
The 3D picture of the absolute error in localization.

In Figure 6, the seven curves that present absolute errors under different radius almost coincide. That means, as long as lengths of step are same, the absolute error is changeless although the radius is different. We can explain this phenomenon as follows: though radius is related to the deployment of virtual beacon, it does not influence the trusty coverage of mobile beacon in the total space. In another word, the change of radius cannot effect whether unknown nodes are covered in the trusty communication extension as long as the step of movement is definite. From Table 2, we can discover that the absolute error is increasing when *R* increases. However, the influence of radius to the error is negligible relative to that of step length. We depict the relationship among radius, length of step, and the absolute error in a 3D picture. In Figure 8, the *x*-label and the *y*-label present radius and length of step. And the *z*-label presents the absolute error of the localization. From the piecemeal change of the color in Figure 8, we find that the influence of step length to the absolute error is greater than that of radius.

### 5.2. The Radius and the Normalized Error

**Figure 9:**
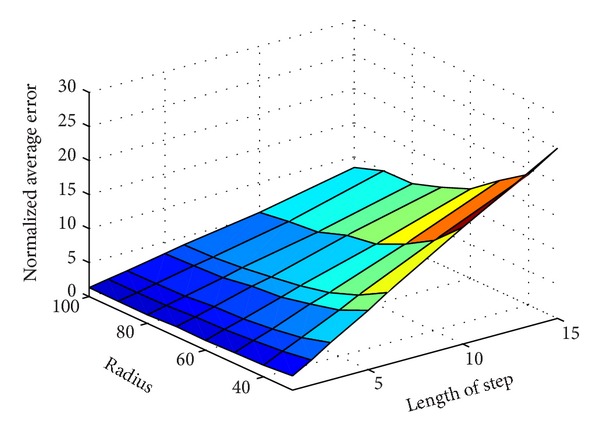
The 3D picture of the normalized error in the localization.

The relationship between radius and the normalized error is shown in Figure 6 and Table 3. Different from Figure 6, we find that the normalized error is decreasing when the radius increases. The 3D picture of the normalized error is shown in Figure 9.

Normalized error is the ratio of the absolute error to the radius. As the absolute error increases, the radius increases. This phenomenon leads to a problem of balancing between radius and accuracy. In localization of WSN, there are two principle elements to be paid attention to: (1) the cost; (2) the accuracy and the precision [40]. In our scheme, the cost is reflected by the radius to a certain extent. Communication cost is influenced by two points: first, the size of model, which affects the number of the packages sent. Second, it is the transmitting power. And these two points are all relative to the radius. Next, we will analyze the relationship among these elements. Take moving step of 1 m for an example, the relationship between radius and number of packages sent is shown in Table 4.

According to the formula of the signal space loss,

(8) $$\begin{array}{c} \left[\text{Lfs}\right]\left(\text{dB}\right)=32.44+20\text{lg }d\;\left(\text{km}\right)+20\text{lg }f\;\left(\text{MHz}\right). \end{array}$$

The following equations should be established:

(9) Lfs(d)=32.44+20lg⁡(0.03)+20lg f+20lg⁡(d0.03)=Lfs(0.03)+20lg⁡(d0.03).

In the light of the operating frequency = 2.4 GHz and the receiving sensitivity = −105 dBm of the CC2420 receiver, and the transmitting power is *W*_1_ = −35 (dBm) when the radius is 30 m, the transmitting powers under different radius are shown in Table 5.

The formula of transforming dBm to mw obeys this equation:

(10) x (dBm)=10lg⁡[p (mw)].

Combined with Table 4, we show the ratio of consumed energy under the same step length in Table 6.

As described in Table 6, it is obvious that the consumed energy is lowest when the radius is 30 m. So, the performance is increasingly excellent as the radius is reducing. At this moment, the traditional normalized error is meaningless. However, there is a drawback that the trajectory is less controllable in the short-radius localization of our scheme.

### 5.3. The Step Length and the Absolute Error

Taking *R* = 100 m for an example, the relationship between moving step and number of packages send is shown in Table 7. 

According to the ratio of the message package numbers, we can get the approximate ratio of the consumed energy with different step length. Because the consumed energy of every package is equal, the product of absolute error and number of the package can be a norm to reflect the relative error of the localization under the same radius but with different step lengths. When *R* = 100 m, the relative error is shown in Table 8. 

On the basis of Table 8, we find that the relative error is the lowest when the step length is 10 m. So, 10 m is the best length of steps to get the proper accuracy under relative smaller overhead of communication when *R* = 100 m.

## 6. The Error Analysis

The error in the process of localization is an important criterion of the locating performance. The goal of error analysis is finding out the “source of error” in order to improve and optimize the schemes in the future work. It is usually that errors are caused by various elements. In this section, we will analyze the main element that leads to the ultimate error of the localization. In our scheme, the largest error is aroused in the process of least square method. The reason is that the referenced point may not be the exact foot point of the trajectory. The error starts at the selection of reference point. Assume that, the length of moving step is *a*, the distance between the foot of the trajectory's perpendicular and the reference point  Δ  is in the interval [0, *a*/2]. If the coordinates of the reference points are

(11) (xa,ya,za)(xb,yb,zb)(xc,yc,zc)

and the coordinates of the foot of the trajectory's perpendicular are

(12) (xa′,ya′,za′)(xb′,yb′,zb′)(xc′,yc′,zc′).

As described in Section 3, the direction vectors are

(13) (i1,j1,k1)=(1,3,0),(i2,j2,k2)=(−1,3,0),(i3,j3,k3)=(0,0,1).

So, (3) is converted into

(14) (xyz)=(12−12036360001)(xa+3ya−xb+3ybzc)

(15)   ⇒(xyz)=((xa+xb3ya−3yb)23(xa−xb+3ya+3yb)6zc).

As to the specific size of the model, the relationship between the foot of the trajectory's perpendicular and the reference point is revealed as follows:

(16) $$\begin{array}{c} \left(\begin{array}{ccc} x_{a} & y_{a} & z_{a}\\ x_{b} & y_{b} & z_{b}\\ x_{c} & y_{c} & z_{c} \end{array}\right)=\left(\begin{array}{ccc} x_{a}^{\prime }+\frac{\Delta }{2} & y_{a}^{\prime }+\frac{\sqrt{3}\Delta }{2} & z_{a}\\ x_{b}^{\prime }+\frac{\Delta }{2} & y_{b}^{\prime }-\frac{\sqrt{3}\Delta }{2} & z_{b}\\ x_{c} & y_{c} & z_{c}^{\prime }+\Delta \end{array}\right). \end{array}$$

Equation (15) should be transformed into

(17) (xyz)=((xa′+xb′+3ya′−3yb′)2+2Δ3(xa′−xb′+3ya′+3yb′)6zc′+Δ).

According to the above, the ultimate error is

(18) (2Δ)2+(Δ)2=5Δ,

owing to the

(19) $$\begin{array}{c} \Delta \in \left[0,\frac{a}{2}\right], \end{array}$$

the theoretical maximum ultimate error

(20) max⁡(errorabsolute)=52a,max⁡(errornormalized)=5a2R.

In Table 2, we discovered that a few of errors are greater than the theoretical value as shown in Tables 9 and 10. This is caused by the imperfection of the model. The balance of size of the space and the model is also a key point of the localization here.

## 7. Conclusion and Future Work

HL is a range-free localization scheme and can be applied under the case that the hardware is relatively limited. We use the special property of trajectory's perpendicular to calculate the coordinate of the unknown nodes. The trajectory is optimized via the geometry constraint, and the locating process is simulated by the tool of MATLAB. The performance of HL is relative perfect in aspect of accuracy. We compare the accuracy under different radius and obtain the coarse bound of the suitable radii. However, the mobile beacon's trajectory may be uncontrollable, and the trajectory may not be the ultimate one. In the next period of work, we shall devote ourselves to studying in the research of model optimization.

## Figures and Tables

**Table 1:** The trusty radius of different radii.

Radius (m)	30	40	50	60	70	80	90	100
Trusty radius (m)	20	25	30	35	40	45	50	60

**Table 2:** The absolute error (m) under different radii and lengths of step.

*R* (m)	Step (m)						
	1	2	3	5	6	10	15
30	0.63144	1.1046	1.6426	2.6104	3.1706	5.1427	7.5045
40	0.68496	1.2053	1.7034	2.7165	3.2887	5.2988	7.6834
50	0.7947	1.3097	1.7838	2.866	3.3216	5.3687	8.1604
60	0.85757	1.3753	1.843	2.8639	3.4115	5.275	7.8843
70	1.0429	1.5191	2.035	3.0941	3.5115	5.6858	7.9872
80	0.99774	1.5054	1.9853	2.9654	3.5361	5.3063	7.9795
90	1.1535	1.6372	2.1323	3.1556	3.5957	5.5929	8.1876
100	1.3238	1.831	2.2867	3.3089	3.8477	5.5941	8.424

**Table 3:** The normalized error under different radii and length of step.

*R* (m)	Step (m)						
	1	2	3	5	6	10	15
30	2.1048	3.682	5.4753	8.7014	10.569	17.142	25.015
40	1.7124	3.0133	4.2585	6.7911	8.2217	13.247	19.209
50	1.5894	2.6193	3.5677	5.732	6.6431	10.737	16.321
60	1.4293	2.2922	3.0717	4.7731	5.6858	8.7917	13.14
70	1.4899	2.1701	2.9071	4.4201	5.0164	8.1226	11.41
80	1.2472	1.8818	2.4816	3.7068	4.4201	6.6329	9.9744
90	1.2816	1.8191	2.3692	3.5062	3.9952	6.2144	9.0973
100	1.3238	1.831	2.2867	3.3089	3.8477	5.5941	8.424

**Table 4:** Relationship between radius and number of the packages sent.

Radius (m)	Number of the packages
30	13224
40	9075
50	4888
60	3414
70	3882
80	4350
90	2348
100	1282

**Table 5:** The transmitting powers under different radii (step length = 1 m).

Radius (m)	Transmitting power (dBm)
30	−35.4134
40	−32.9146
50	−30.9764
60	−29.3928
70	−28.0538
80	−26.8940
90	−25.8709
100	−24.9558

**Table 6:** The ratio of consumed energy under the same step length.

Radius (m)	Consumed energy
30	3.8021
40	4.6386
50	3.9038
60	3.9263
70	6.0768
80	8.8938
90	6.0759
100	4.0955

**Table 7:** Relationship between moving step and number of the packages sent (*R* = 100 m).

Step length (m)	Number of the packages
1	1282
2	641
3	427
5	257
6	214
10	128
15	86

**Table 8:** The relative error under different step lengths (*R* = 100 m).

Step length (m)	Relative error
1	1697.1
2	1173.7
3	976.42
5	850.39
6	823.41
10	716.04
15	724.46

**Table 9:** The theoretically absolute error under different length of step.

Step length (m)	Absolute error (m)
1	1.1180
2	2.2361
3	3.3541
5	5.5902
6	6.7082
10	11.1803
15	16.7705

**Table 10:** The theoretically normalized error under different radii and lengths of step.

*R* (m)	Step (m)						
	1	2	3	5	6	10	15
30	3.73	7.45	11.18	18.63	22.36	37.27	55.90
40	2.80	5.59	8.39	13.98	16.77	27.95	41.93
50	2.24	4.47	6.71	11.18	13.42	22.36	33.54
60	1.86	3.73	5.59	9.32	11.18	18.63	27.95
70	1.60	3.19	4.79	7.99	9.58	15.97	23.96
80	1.40	2.80	4.19	6.99	8.39	13.98	20.96
90	1.24	2.48	3.73	6.21	7.45	12.42	18.63
100	1.12	2.24	3.35	5.59	6.71	11.18	16.77
